# Supplementary material for: Ultrathin ferrite nanosheets for room-temperature two-dimensional magnetic semiconductors
Source: Nat Commun. 2022 Sep 6;13:5241. doi: 10.1038/s41467-022-33017-1 (PMC9448765; doi:10.1038/s41467-022-33017-1)
Supplement: Supplementary file 1 — Supplementary Information [file 41467_2022_33017_MOESM1_ESM.pdf]

Supplementary Information

Ultrathin ferrite nanosheets for room-temperature two-dimensional magnetic semiconductors

Cheng et al.

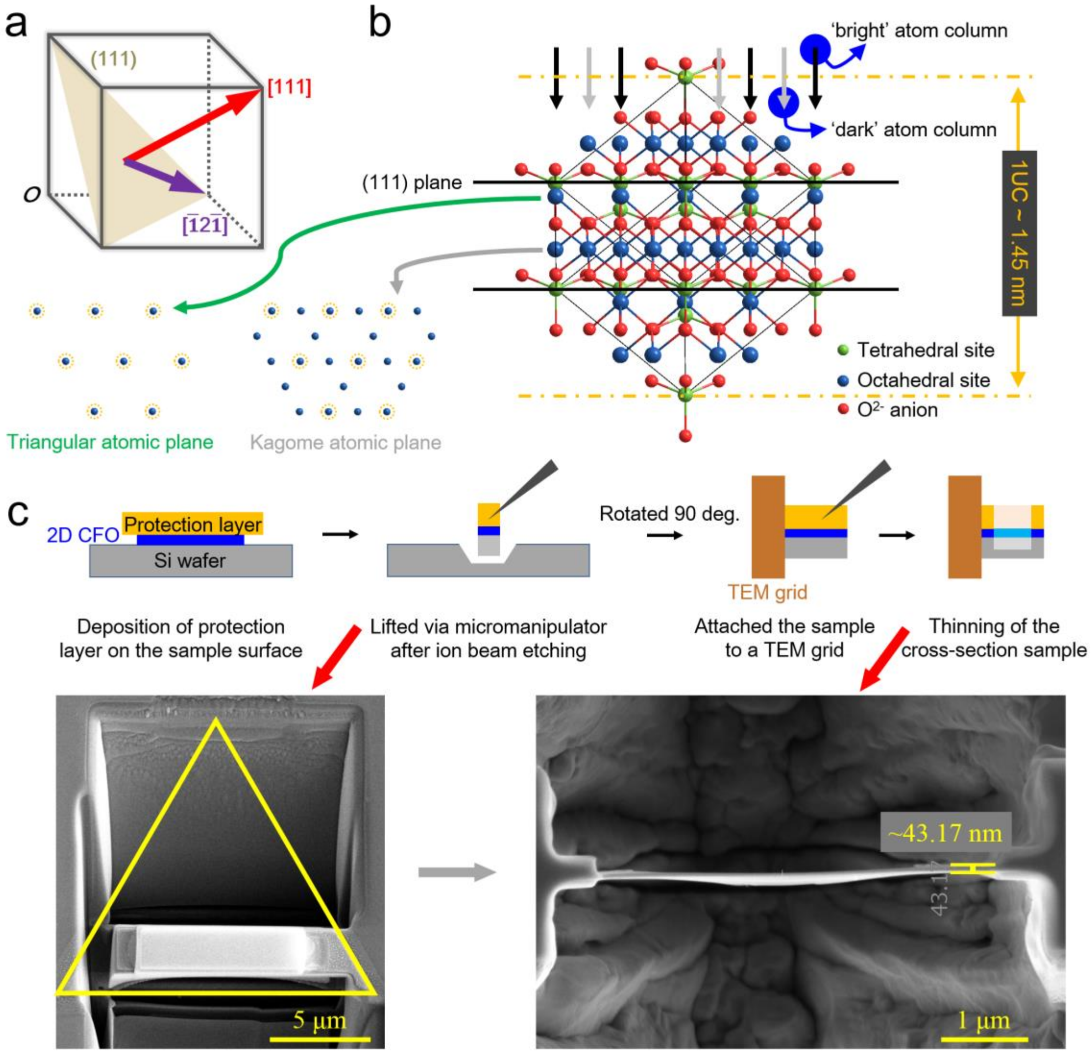

Supplementary Fig. 1 | Atomic model of CFO and SEM images of the sample during FIB milling. a Schematic of a (111)-oriented CFO crystal structure. **b** The side view of CFO (111) lattice planes, revealing its nonlayered structure. The octahedral cations sublattice of CFO nanosheet along the vertical direction consists of alternating triangular and Kagome (made of corner-sharing triangles) atomic planes. **c** SEM images of the sample during Focused Ion Beam (FIB, FEI Helios Nanolab 600i) milling and the corresponding flow diagram. For the TEM cross-section sample preparation, the sectioning line is almost perfectly parallel to one side of the triangular sample (yellow triangle as a schematic diagram, the CFO sample is transferred onto 300-nm SiO_2/Si substrate). The final cross-section sample thickness is around ~43 nm.

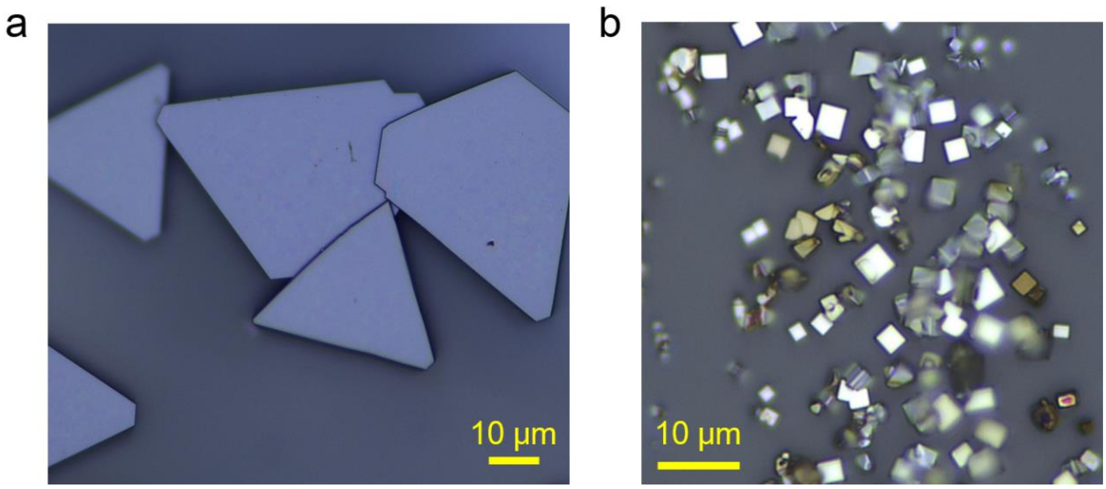

Supplementary Fig. 2 | Control experiments on the sample synthesis. a Only disorderly stacked thick flakes were obtained without molecular sieves keeping the same of other experimental conditions. **b** Large-density, thick cubic islands were obtained when high-purity 15%-H_2/Ar mixture (150 sccm) was used as the carrier gas.

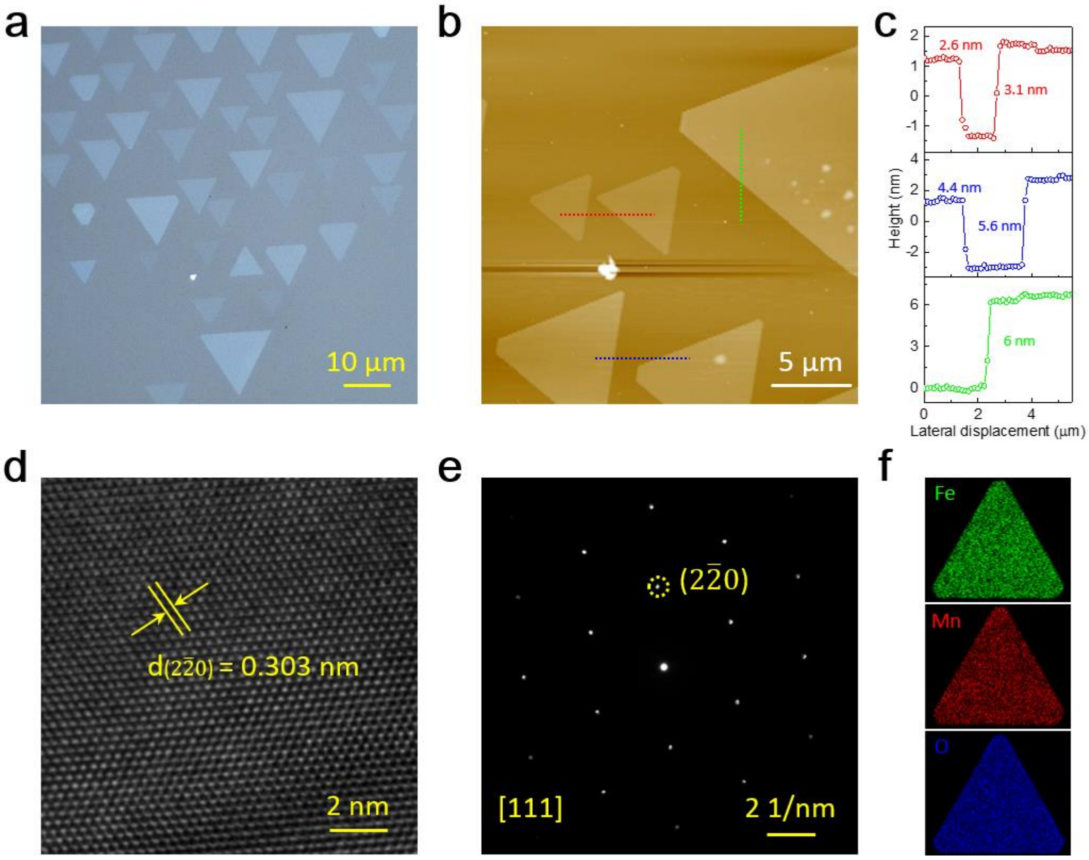

Supplementary Fig. 3 | Van der Waals epitaxial growth of manganese ferrite nanosheets. a OM image of the ultrathin triangular manganese ferrite ($MnFe_2O_4$, MFO) nanosheets. **b, c** AFM image and the corresponding height profiles of MFO nanosheets. **d-f** HRTEM image, SAED pattern and TEM-EDS elemental mapping images of MFO nanosheet. Here the lattice spacing is measured to be 0.303 nm, corresponding to its $(2\bar{2}0)$ planes.

For the synthesis, weighed amount of ferric oxide (α-Fe_2O_3, 99.5%, Aladdin), manganous oxide (MnO, 99.99%, Aladdin), ferric chloride ($FeCl_3$, 99.99%, Sigma Aldrich) and sodium chloride powders with the molar ratio of 1:1:0.2:0.01 were mixed evenly and used as precursors. Other growth condition is the same as that for CFO.

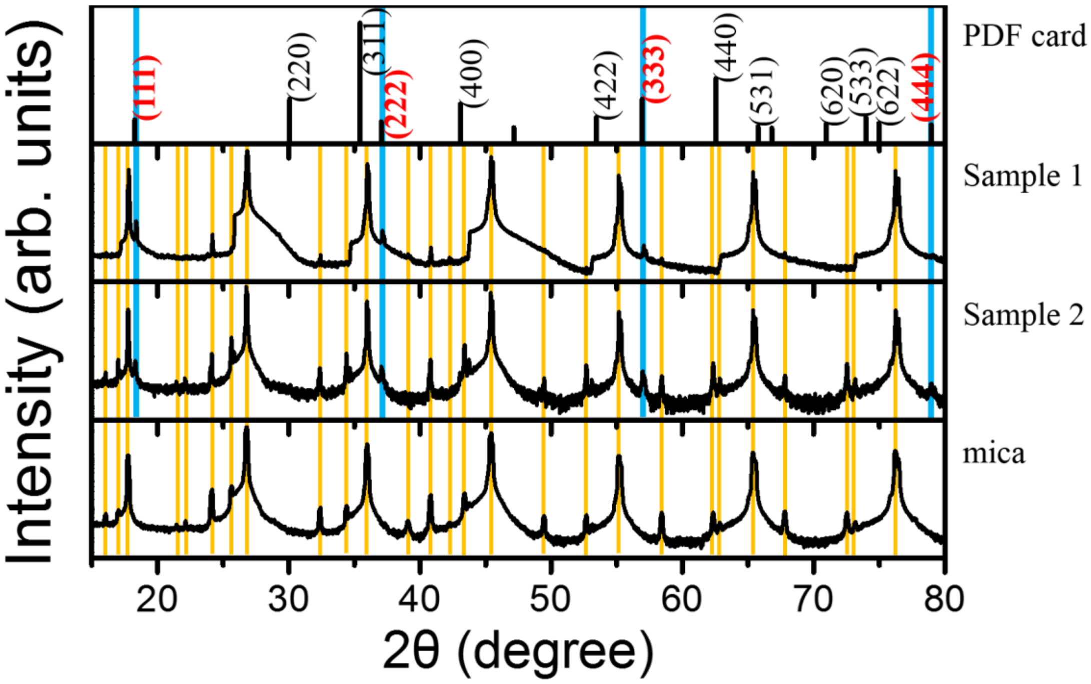

Supplementary Fig. 4 | X-ray diffraction (XRD) spectra of CFO nanosheet samples on the mica substrate. The XRD peaks around 18.3 °, 37.1 °, 57.0 °and 79.0 ° are corresponding to its (111), (222), (333) and (444) planes, confirming the epitaxial nature with (111) preferred orientation.

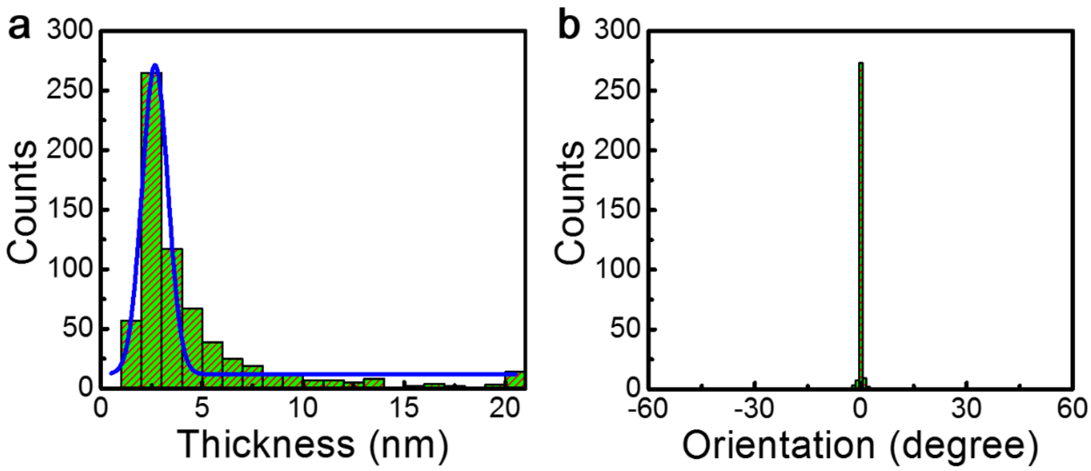

Supplementary Fig. 5 | Histogram statistics of the thickness (a) and orientation (b) of CFO nanosheets with substrate temperature of 700 ℃, smooth curve is the Gaussian fit of the thickness distribution. The resulting nanosheets exhibit a relatively narrow thickness distribution (~2-4 nm) and nearly identical crystallographic orientations.

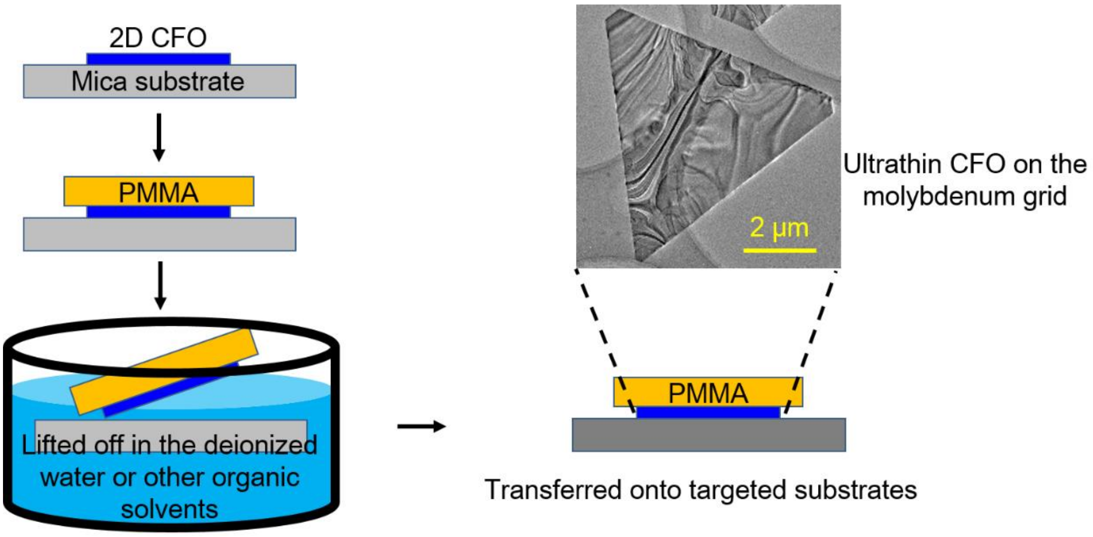

Supplementary Fig. 6 | Scheme of transferring CFO nanosheet from mica onto targeted substrates. The well-preserved morphology after transfer confirms the stability and transferability of ultrathin CFO nanosheets.

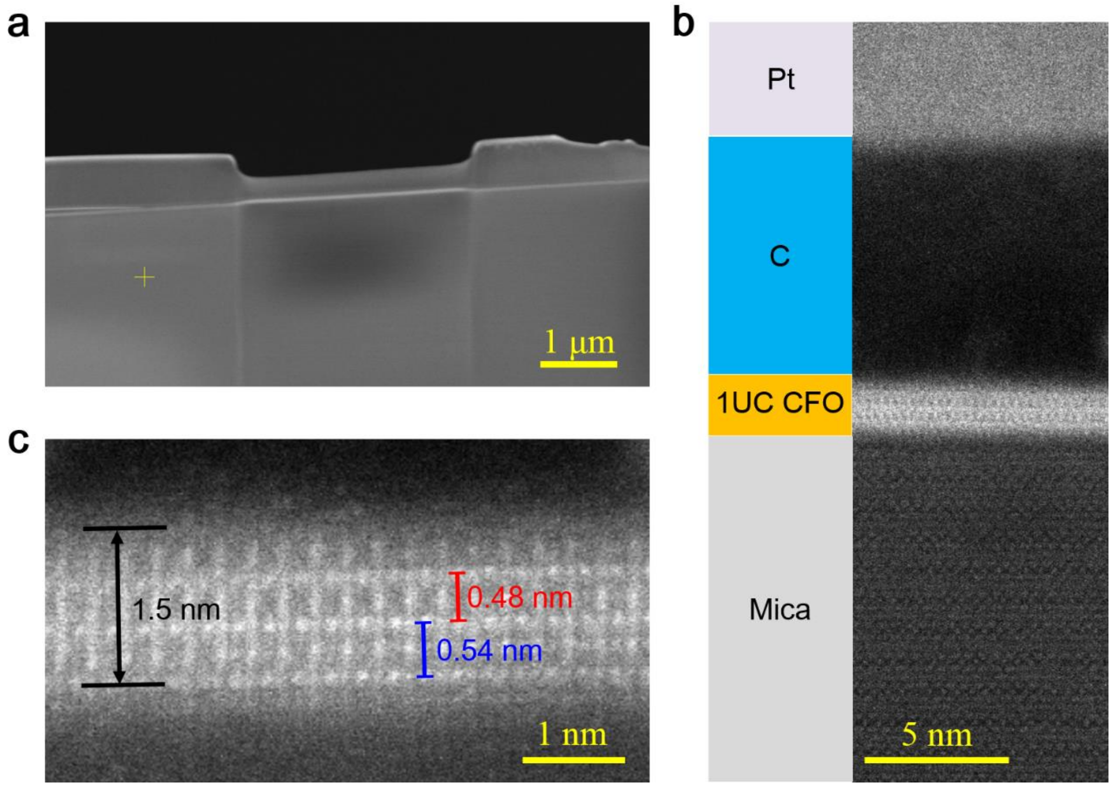

Supplementary Fig. 7 | Cross-sectional HAADF-STEM image of 1-unit-cell CFO nanosheet. a SEM image of the 1-unit-cell CFO nanosheet sample prepared by Focused Ion Beam milling. **b, c** Cross-sectional HAADF-STEM image of 1-unit-cell CFO nanosheet, which shows a periodic rectangular pattern in the vertical direction, corresponding to three subcells with different atomic distributions.

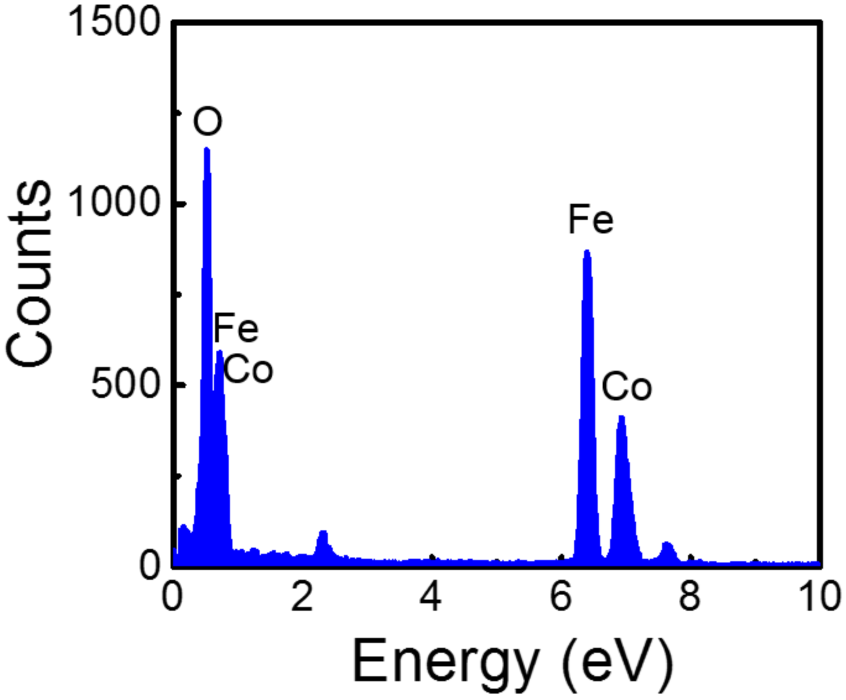

Supplementary Fig. 8 | The atomic percentage ratio of Co, Fe and O is approximately 0.97:2.11:4, consistent with the expected 1:2:4 ratio for $CoFe_2O_4$. The minor deviation may come from the experimental error and the possible loss of O under electron irradiation. Besides, although chlorides is introduced during the growth process, the atomic ratio of Cl element in the CFO nanosheet is 0.00%.

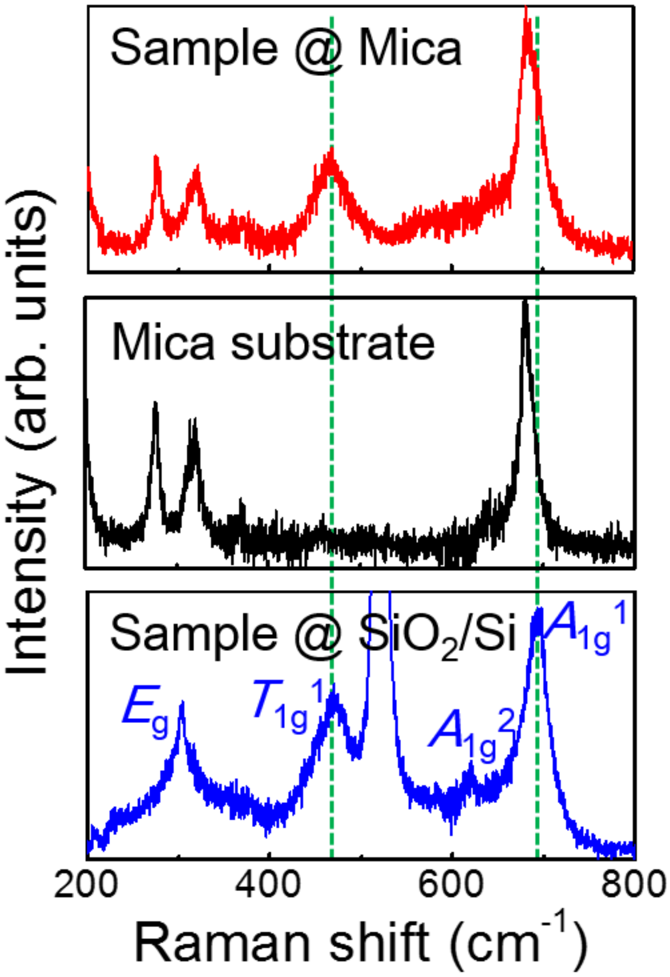

Supplementary Fig. 9 | Raman spectra of CFO nanosheets grown on mica substrate (top), pure mica substrate (middle) and CFO nanosheet transferred onto SiO_2/Si substrate (bottom).

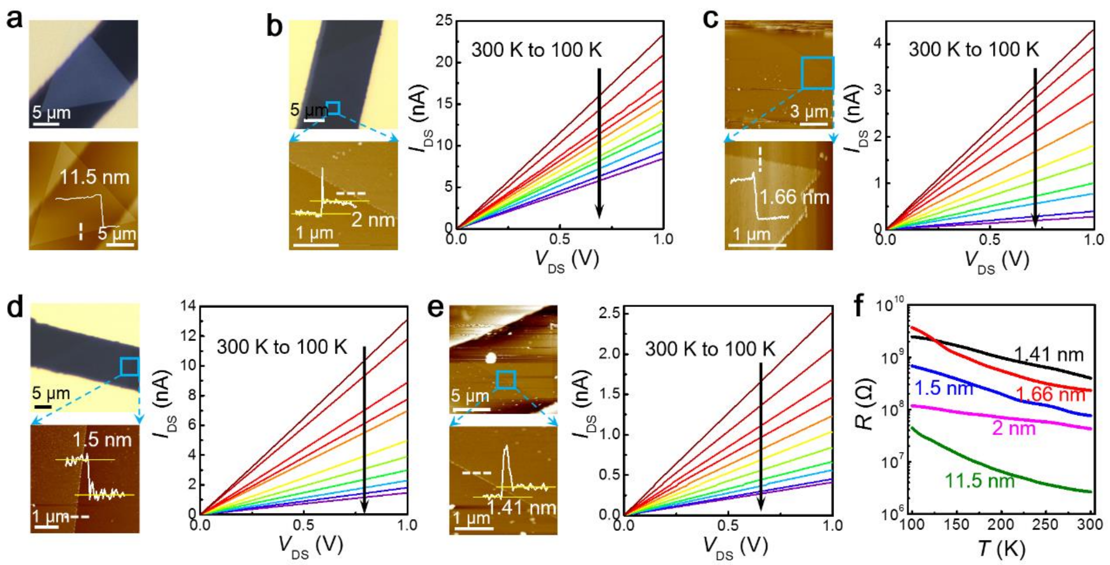

Supplementary Fig. 10 | Temperature-dependent electrical measurements of CFO nanosheets. a OM (up) and AFM (down) images of the device studied in the manuscript. **b-e** Temperature-dependent electrical measurements of CFO nanosheets with 1-unit-cell thickness. The corresponding device morphology and height profiles are shown in the inset. The linear I_{DS}–V_{DS} characteristics indicate the devices have good Ohmic contact, thus the influence of metal contact can be neglected. **f** Temperature-dependent resistance of CFO nanosheet devices with various channel thicknesses.

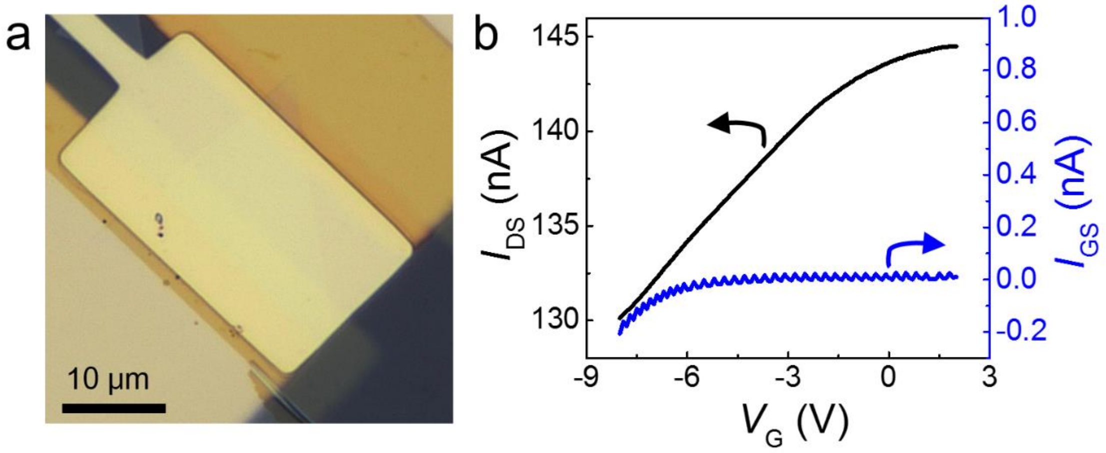

Supplementary Fig. 11 | Room-temperature transfer curve of a representative top-gate CFO device, displaying n-type transistor behavior. V_{DS} was set as 1 V. The leakage current is shown by the blue curve.

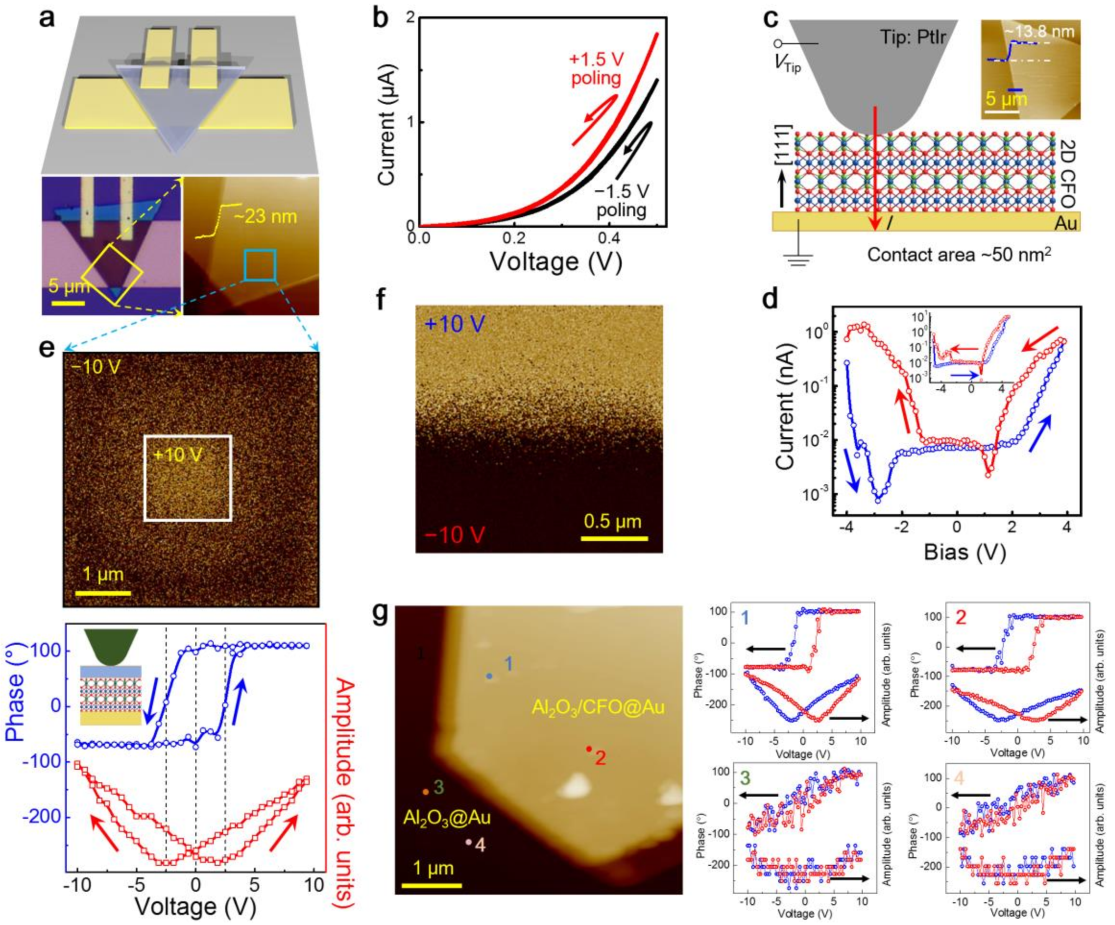

Supplementary Fig. 12 | Electric switching properties of CFO nanosheets. a Structure schematic, OM image and AFM image of a fabricated CFO vertical device. The thickness of CFO nanosheet was determined to be ~23 nm. **b** *I*–*V* curves of CFO nanosheet vertical device obtained after poling with ±1.5 V bias. **c** Schematic of the CAFM set-up with a probe tip mimicking a nanosized PtIr/CFO/Au asymmetric metal–semiconductor–metal (MSM) device structure. The effective contact area is around 50 nm^2. Top inset, AFM image of ~13.8 nm-thick CFO nanosheet on Au coated silicon substrate. **d** *I*–*V* sweep of ~52 nm-thick CFO nanosheet, showing clear hysteresis that resembles typical resistive memories. Top inset, *I*–*V* sweep of ~13.8 nm-thick CFO nanosheet. **e** Upper, PFM phase image of CFO nanosheet obtained after poling with ±10 V bias using the MOS structure. Lower, local PFM phase and amplitude versus voltage hysteresis loops, showing clear ferroelectric polarization switching under external electric field. **f** PFM phase image of ~96 nm-thick CFO obtained after poling with ±10 V bias using the MOS structure. **g** Local PFM phase and amplitude versus voltage hysteresis loops of ~156 nm-thick Al_2O_3/CFO/Au MOS structure and Al_2O_3/Au structure.

For MSM device structure, CFO nanosheet is easily destroyed under the action of high voltages needed for realizing polarization effectively. Thus, we construct a metal–oxide–semiconductor (MOS) structure via introducing 8 nm-thick Al_2O_3 layer, as suggested by Ye et al (*Nat. Electron.* 2019, 2, 580), and perform typical PFM study

on it. However, although Al_2O_3/CFO/Au MOS structure and Al_2O_3/Au structure exhibit distinctly different PFM results, other contributions such as interface charging effect, substrate signals and species diffusion should not be excluded.

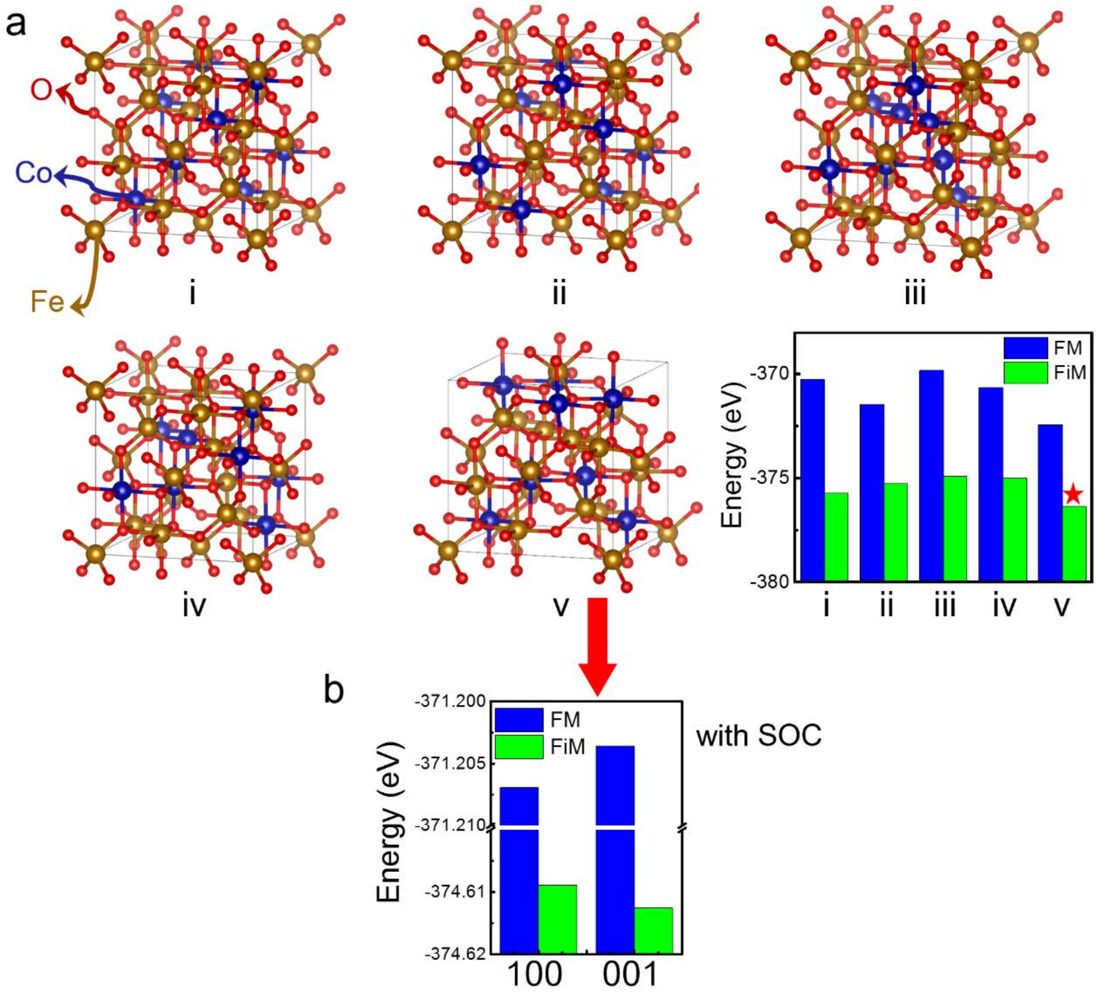

Supplementary Fig. 13 | The magnetic structures of CFO. a Cation distribution of Fe and Co for five different configurations used in our calculations. Eight Fe cations occupying the tetrahedral sites, both eight Co and eight Fe cations occupying the octahedral sites. To determine the ground-state energy, we considered the ferromagnetic (FM) as well as ferrimagnetic (FiM, ferromagnetic Néel type) structures, where the magnetic moments of tetrahedral and octahedral cations are aligned in a parallel and antiparallel manner, respectively. On the one hand, configuration v is lower in energy than other configurations, whether it is FM or FiM structures. On the other hand, the energy of FiM structures is lower than the corresponding FM structures. **b** Energy comparison between FM and FiM structures of configuration v along different directions by considering the spin-orbit coupling. The energy of FiM structure is significantly lower than the corresponding FM structure.

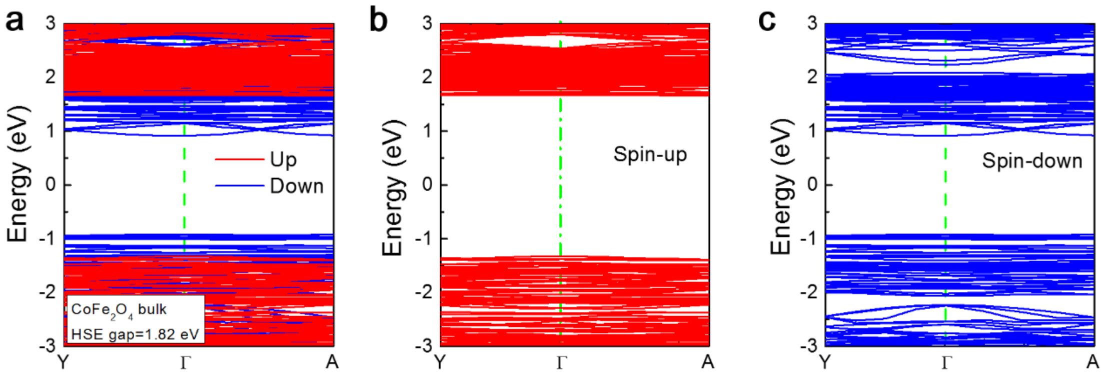

Supplementary Fig. 14 | Calculated electronic band structure of bulk CFO with spin-up (left) and spin-down (right) components using the first principles simulations. The conduction band minimum and valence band maximum are located at the *Γ* point with a bandgap of 2.98 eV for spin-up channel and 1.82 eV for spin-down channel, suggesting the magnetic band structure of CFO with spin-splitting. Fermi energy is set to zero.

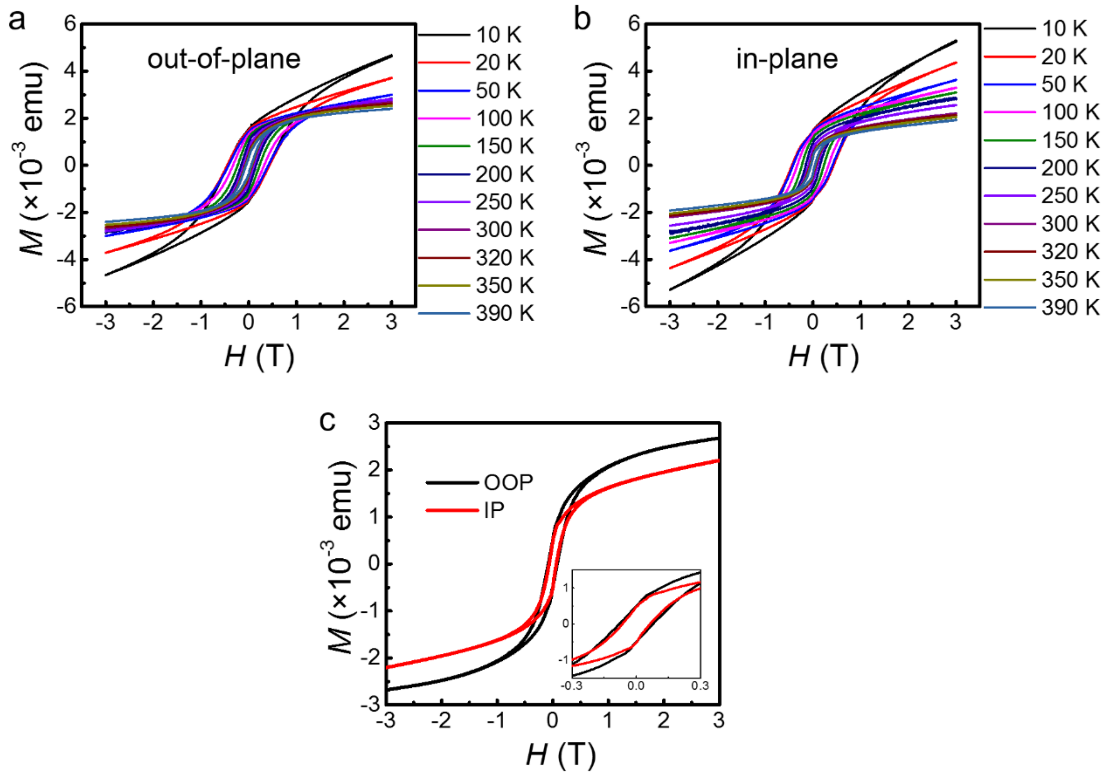

Supplementary Fig. 15 | VSM measurements of CFO nanosheet samples. a, b Magnetic hysteresis loops of CFO nanosheet samples measured at different temperatures under OOP (**a**) and IP (**b**) magnetic field, respectively. The magnetic field is swept between ±3 T. **c** The comparison between *M*-*H* curves under OOP and IP magnetic field at room temperature evidences an OOP magnetic easy axis (i.e. higher magnetization and larger coercive force).

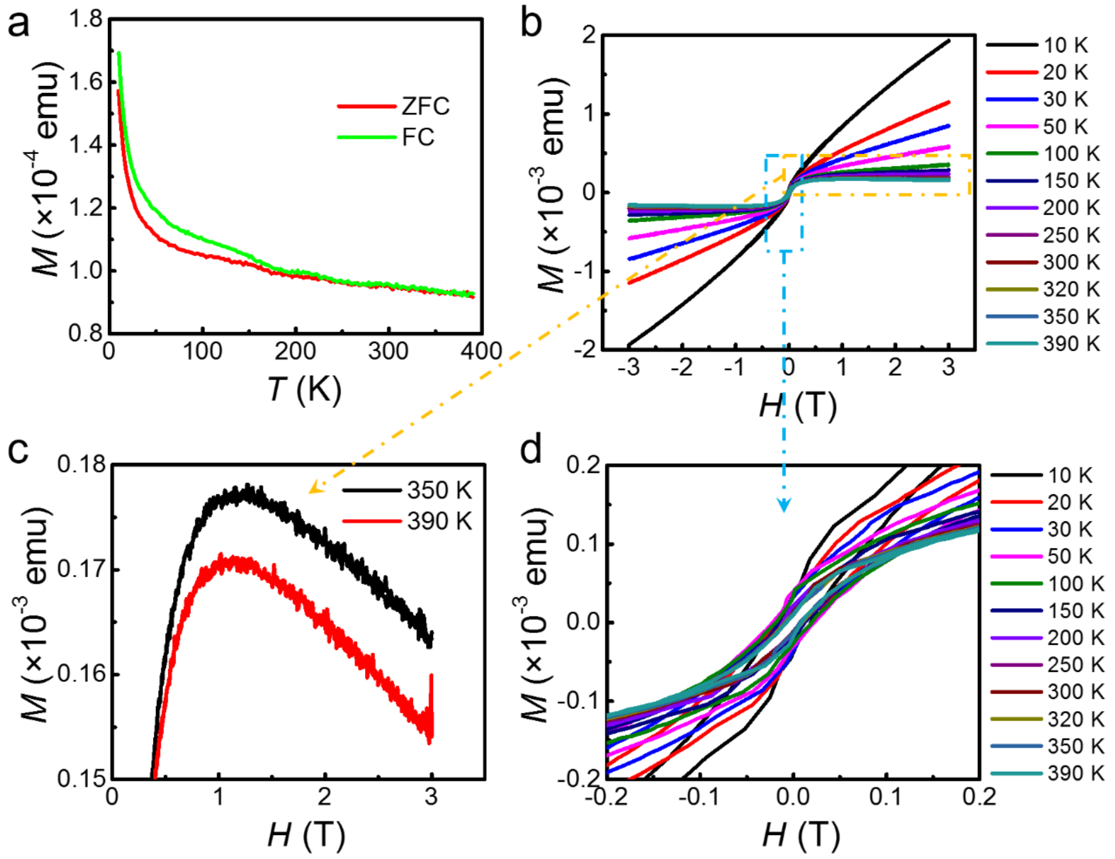

Supplementary Fig. 16 | VSM measurements of the mica substrate. a Temperature dependence of magnetic susceptibility (measured with a field of 0.1 T) of the mica substrate, indicating the sharp increase of magnetization at low temperatures (from 40 to 10 K) can be attributed to the paramagnetism of mica substrate at low temperatures (possibly originated from the impurities). **b-d** Magnetic hysteresis loops of the mica substrate measured at different temperatures. The magnetic field is swept between ±3 T. As shown, the magnetic susceptibility varies linearly with the applied field at high magnetic field. In particular, the magnetic susceptibility decreases linearly with the increasing field at high temperatures, showing typical diamagnetism (**c**). Thus we can conclude that mica substrate exhibits paramagnetism at low temperatures (below 40 K) and diamagnetism at high temperatures (above 300 K). Although the hysteresis loops were observed in the *M*-*H* curves (**d**), they are independent of the temperature and much weaker than the magnetism of CFO samples.

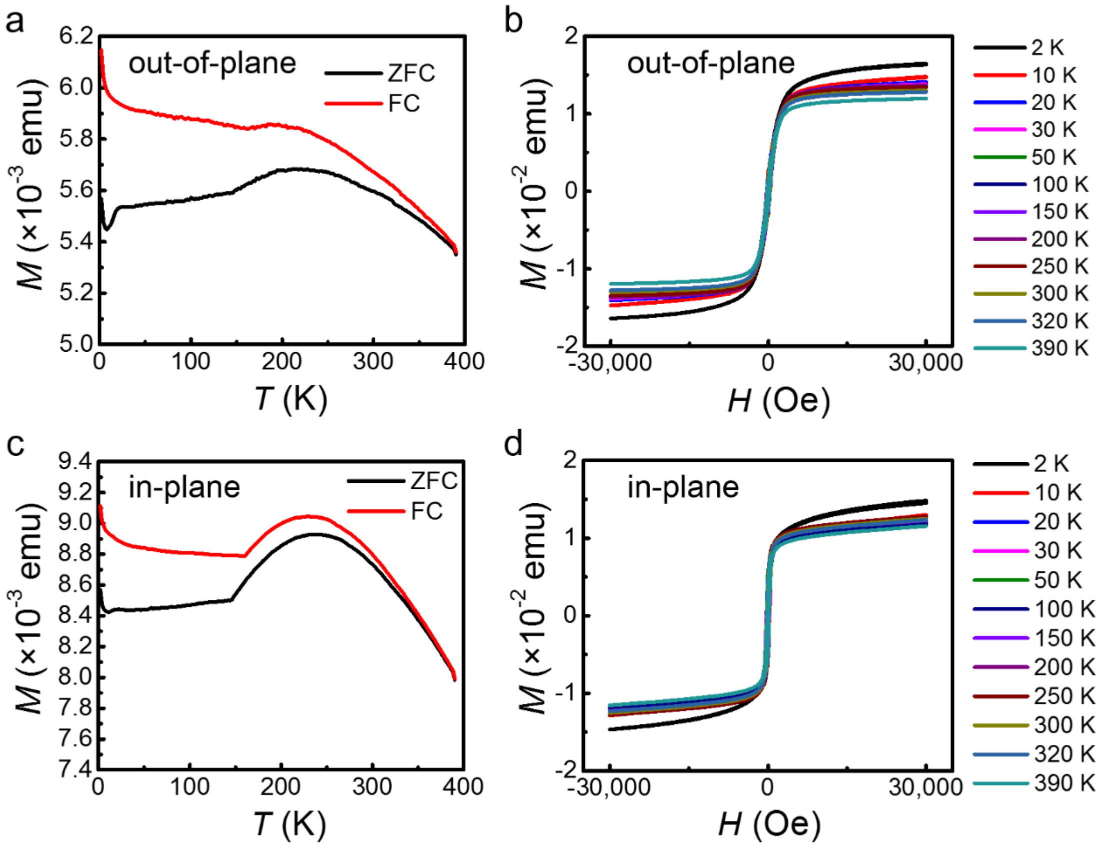

Supplementary Fig. 17 | VSM measurements of manganese ferrite nanosheet samples. a, c Temperature dependence of magnetic susceptibility (measured with a field of 0.1 T) of manganese ferrites nanosheet samples under OOP and IP magnetic field, respectively. **b, d** Magnetic hysteresis loops of manganese ferrites nanosheet samples measured at different temperatures under OOP and IP magnetic field, respectively. VSM results demonstrated that manganese ferrite nanosheets is a soft magnetic material.

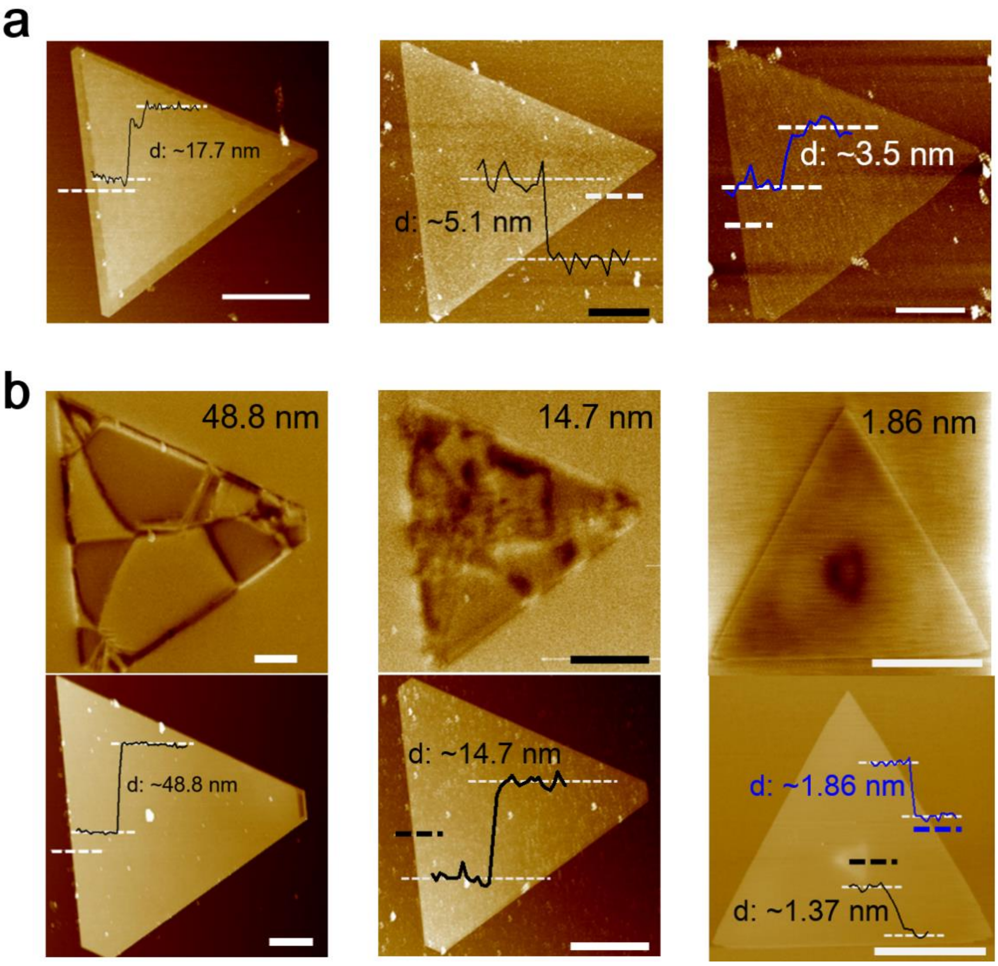

Supplementary Fig. 18 | MFM investigation of CFO nanosheets. a Topographic images of CFO nanosheets studied in the manuscript. Scale bars: 3 μm. **b** MFM phase images and AFM images of CFO nanosheets with variable thicknesses. Scale bars: 3 μm.

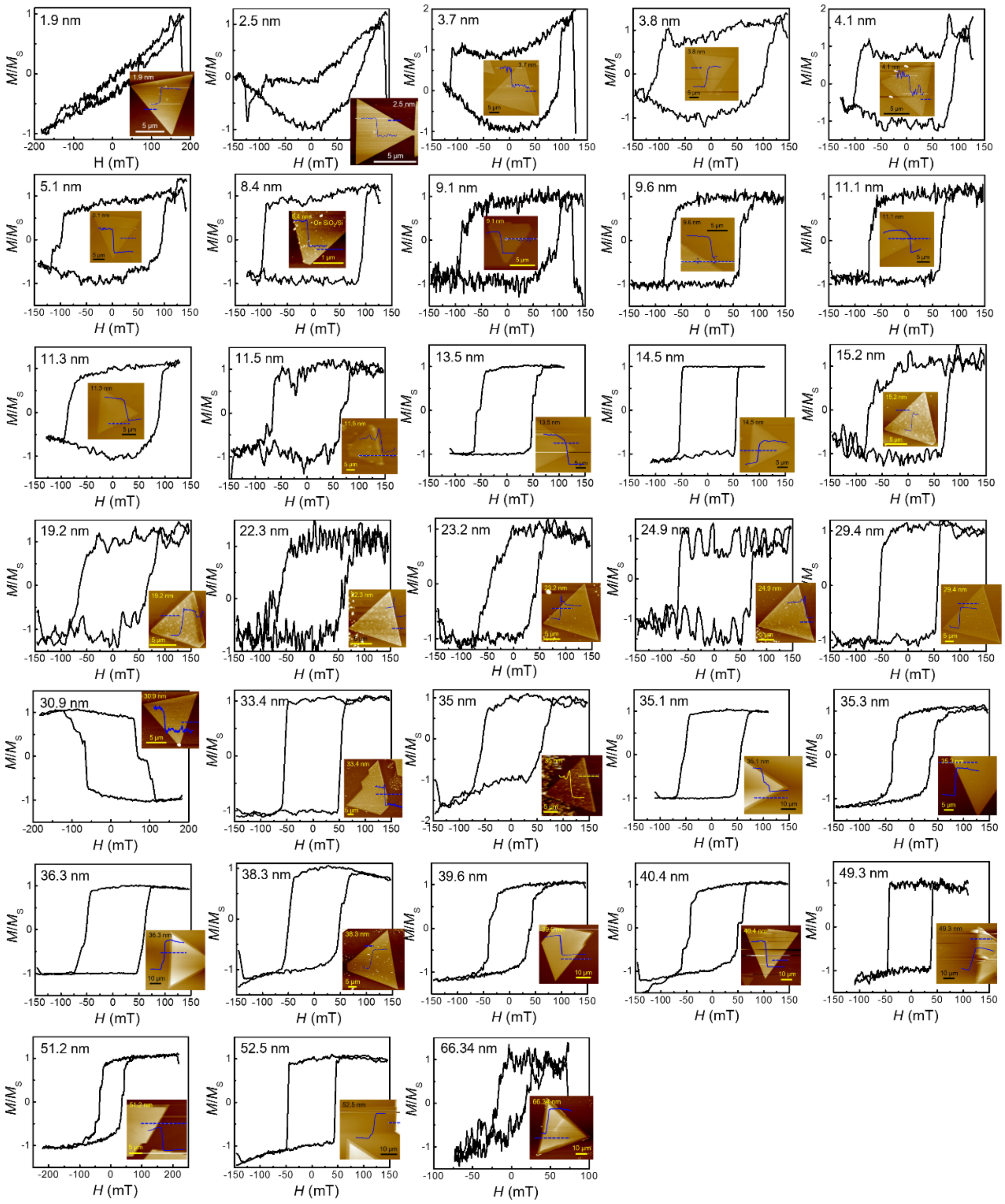

Supplementary Fig. 19 | Room-temperature MOKE hysteresis loops of 33 CFO nanosheets with variable thicknesses under OOP magnetic field. Note that different substrates (mica and silicon) are adopted for MOKE measurement, suggesting that the magnetic properties are intrinsic and CFO nanosheets can be regarded as isolated magnets.

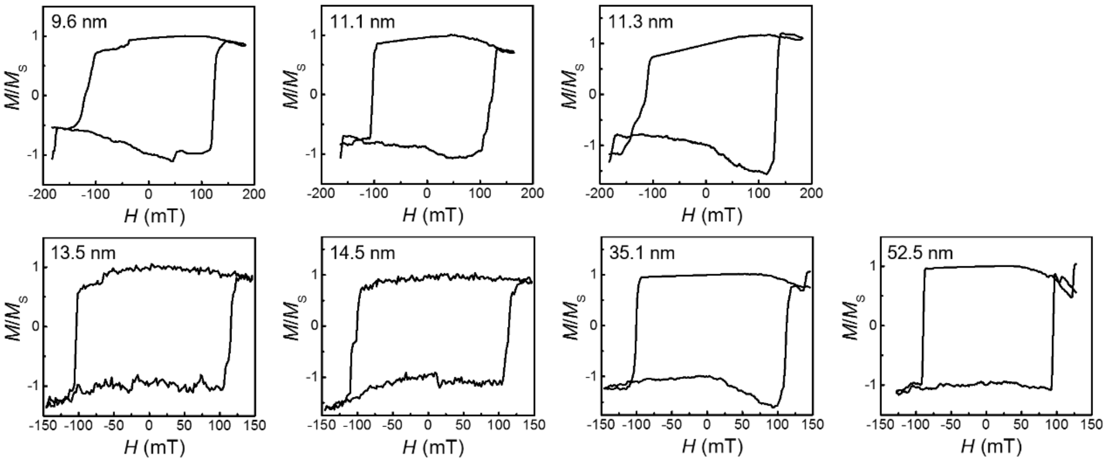

Supplementary Fig. 20 | Magnetic properties of CFO nanosheets at cryogenic temperature: MOKE hysteresis loops of CFO nanosheets with variable thicknesses at 80 K.

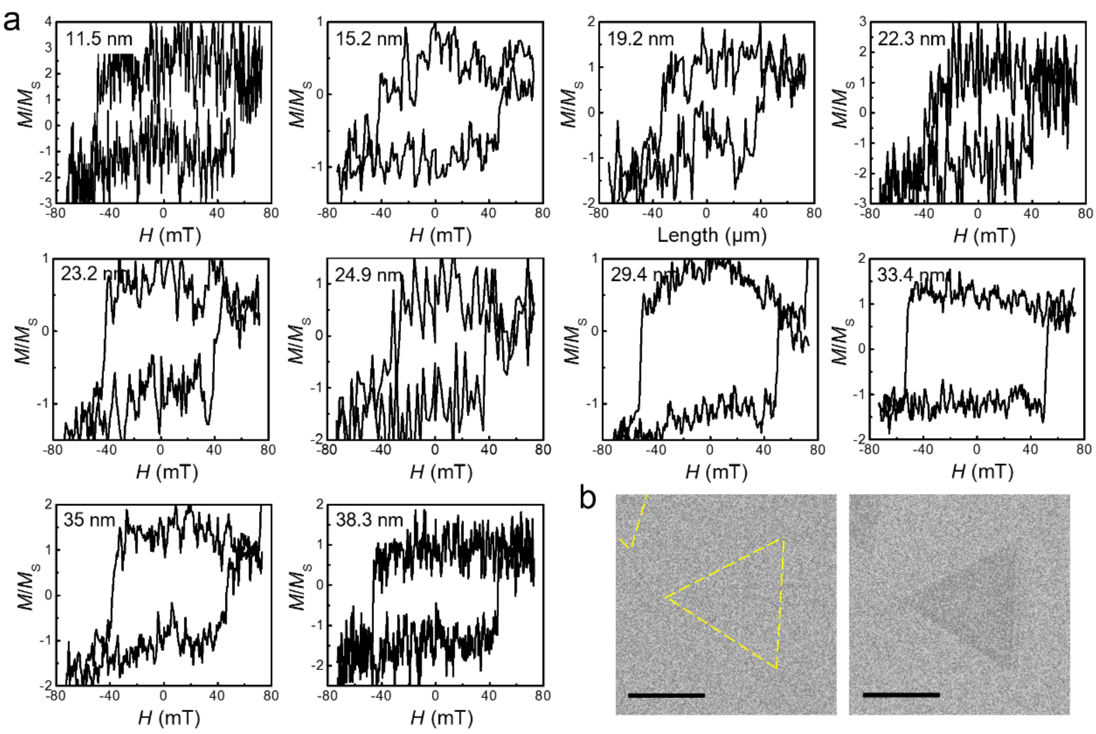

Supplementary Fig. 21 | Magnetic properties of CFO nanosheets at elevated temperature. a MOKE hysteresis loops of CFO nanosheets with variable thicknesses at 360 K. **b** MOKE microscopy images of a 29.4 nm-thick CFO nanosheet during the magnetization reversal at 360 K. Scale bars: 20 μm.

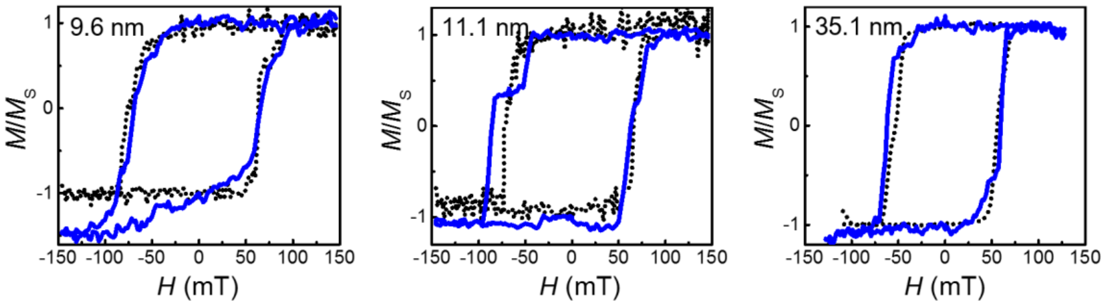

Supplementary Fig. 22 | Air stability investigation of CFO nanosheets without encapsulation. Compared with the initial states (dotted lines), the room-temperature MOKE signal showed little change after exposure to ambient conditions for over a month (solid lines), demonstrating the well air stability of CFO nanosheet.

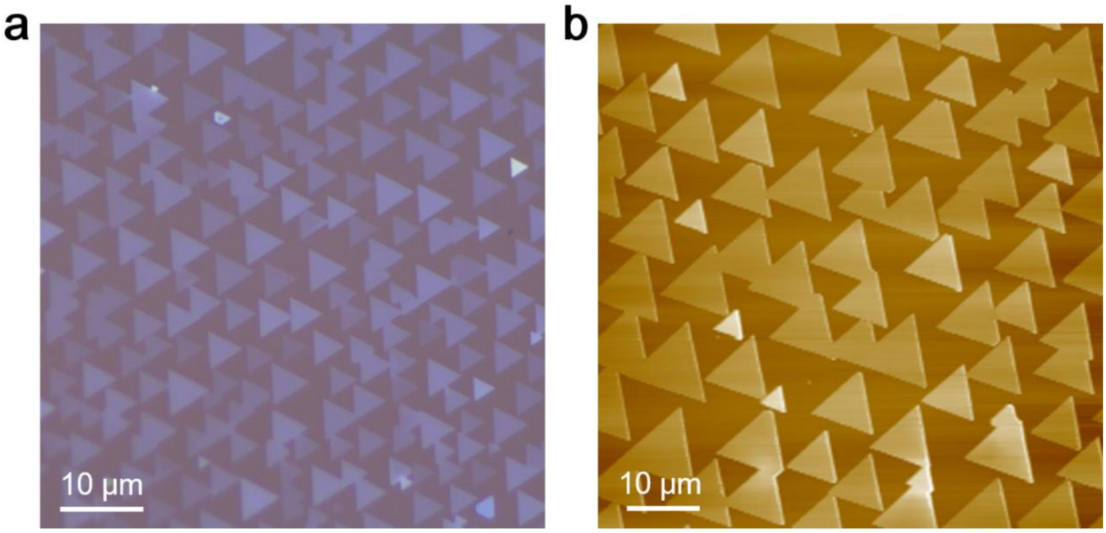

Supplementary Fig. 23 | Topographies of CFO nanosheets by adjusting the growth temperature. Optical microscope image (**a**) and AFM image (**b**) of CFO nanosheets with substrate temperature of 670 ℃, which show higher coverage (up to 70%) but larger thickness (~14-18 nm) compared with the conditions described in the manuscript (700 ℃).
